# The evolution of New Zealand’s health workforce policy and planning system: a study of workforce governance and health reform

**DOI:** 10.1186/s12960-019-0390-4

**Published:** 2019-07-05

**Authors:** Gareth H. Rees

**Affiliations:** ESAN University, Alonso de Molina 1652, Monterrico Chico, 33 Lima, Peru

**Keywords:** Health reform, Health workforce governance, Health workforce policy, Health workforce planning, Workforce innovation, New Zealand

## Abstract

**Introduction:**

While considerable attention has been given to improving health workforce planning practice, few articles focus on the relationship between health workforce governance and health reform. By outlining a sequence of health reforms, we reveal how New Zealand’s health workforce governance and practices came under pressure, leading to a rethink and the introduction of innovative approaches and initiatives.

**Case description:**

New Zealand’s health system was quite stable up to the late 1980s, after which 30 years of structural and system reform was undertaken. This had the effect of replacing the centralised medically led health workforce policy and planning system with a market-driven and short-run employer-led planning approach. The increasing pressures and inconsistencies this approach produced ultimately led to the re-centralisation of some governance functions and brought with it a new vision of how to better prepare for future health needs. While significant gain has been made implementing this new vision, issues remain for achieving more effective innovation diffusion and improved integrated care orientations.

**Discussion and evaluation:**

The case reveals that there was a failure to consider the health workforce in almost all of the reforms. Health and workforce policy became increasingly disconnected at the central and regional levels, leading to fragmentation, duplication and widening gaps. New Zealand’s more recent workforce policy and planning approach has adopted new tools and techniques to overcome these weaknesses that have implications for the workforce and service delivery, workforce governance and planning methodologies. However, further strengthening of workforce governance is required to embed the changes in policy and planning and to improve organisational capabilities to diffuse innovation and respond to evolving roles and team-based models of care.

**Conclusion:**

The case reveals that disconnecting the workforce from reform policy leads to a range of debilitating effects. By addressing how it approaches workforce planning and policy, New Zealand is now better placed to plan for a future of integrated and team-based health care. The case provides cues for other countries considering reform agendas, the most important being to include and consider the health workforce in health reform processes.

## Background

Much of health workforce policy and planning efforts are directed towards matching worker numbers and skills with expected demand using quantitative models [1]. This tends to provide better performance over shorter time periods, and it is therefore more suited to service planning [2]. While the health workforce literature has valuable directions for improving methods, models and accuracy [3–5], less attention has been paid to health policy’s relationships and effects on health workforce development, planning and provision [6]. This is largely due to health policy not adequately considering workforce issues [7] nor the impact on its governance [8], which is the collection of mechanisms, structures, processes and influences for a system’s oversight, policies, planning and accountability [6, 9, 10]. Hence, the article’s aim is to contribute to the nascent literature on this topic, through an analysis of New Zealand’s oscillating pattern of decentralising-recentralising health reforms [11] and their effects on the country’s health workforce and its governance.

Relatively stable to the late 1980s, New Zealand’s health system began experiencing significant and widespread changes driven by the aims of improving access, service integration and overall system efficiency [12]. Summarised in Table 1, the reform progression started from the Department of Health’s (DoH) centralised provision of hospital, public health and mental health services and devolved firstly into regional and then smaller business units to deliver hospital-based care [15], founded a new policy-based Ministry of Health (MoH), provided Public Health through a separate commission and saw the primary care (PC) sector organising into locally based associations. Hospital user fees were trialled and abandoned. In the early 2000s, a new regionalised structure was put into place. This reform established district health boards (DHB) and mechanisms for local input and provided more attention to PC delivery [15, 16]. Regardless, New Zealand’s health system continued to be somewhat fragmented with its models of care and services remaining largely speciality or sectorially aligned [12]. From the late 2000s, system change became more evolutionary and a move towards re-centralisation began [9, 12, 17]. These changes included the introduction of (i) district alliances (DA), a joint planning method to achieve high-level system outcomes [9, 18], (ii) a new system of mixed performance measures [13, 17], (iii) policy dedication to patient-focussed care and community-based provision [19] and (iv), due to recurring and persistent workforce issues throughout the previous reforms, the allocation of responsibility for New Zealand’s health workforce sustainability and planning [11].

**Table 1:** Thirty years of New Zealand health reforms

Policy mode	Centralised	Decentralisation				Recentralisation
Timeframe	Pre mid-1980s	Mid-late 1980s	Early 1990s	Mid-late 1990s	2000s	Late 2000s on
Focus	Universal access	New public management	Efficiency	Value for money	Responsiveness	Performance
Organisation	Centralised and relatively stable	Regionalisation: 23 area units established	Commercialisation: hospital part-charges introduced	Return to free-at-entry services	Democratisation: partially elected District Health Boards (DHB)	DHB and PC alignment Alliance contracting
Means of control	Department of Health manages all facets and payments	Policy Ministry with purchasing entity Regional services through contracts and financial targets	Internal market model Hospitals operated as independent business units	Policy Ministry with 4 regional funder-provider authorities	DHB plans and funding agreements with Ministry; New PC focus	Partial re-centralisation New focus on patient-focussed care

Sources: [9, 11–14]

Thus, the article proceeds by presenting the body of the case description that summarises the impacts and effects of these health policy and system changes have had on New Zealand’s health workforce system. This is followed by a commentary on the implications for the workforce and service delivery, for health workforce governance and for workforce planning methods to address a rapidly changing environment, evolving roles and team-based models of care. The article closes with a short conclusion.

## Case description

### New Zealand’s health system

New Zealand’s health system is framed by the 1938 Social Security Act, which aimed for a universal and free public health service. However, concessions to the PC sector compromised this vision, as the general practitioner (GP) workforce was permitted to operate as independent private businesses and to charge patients, unlike their salaried hospital-based counterparts who provided free-at-entry treatment [15]. This situation, which still exists, contributes to system fragmentation, poor service integration, and funding and governance inconsistencies [12, 15]. Approximately 80% of the country’s health care costs are government funded through tax-paid services, attendance subsidies and compulsory accident insurance payments [11].

### New Zealand’s health workforce policy and planning

New Zealand’s recent health workforce policy and planning has broadly evolved in three phases (see Table 2)

**Table 2:** Three phases of New Zealand’s health workforce policy

Category	Issue	Phase 1	Phase 2			Phase 3
		Pre mid-1980s	Mid-late 1980s	Mid-late 1990s	Early-mid 2000s	Late 2000s on
Implications of reforms for the workforce and service delivery	Focus	Centralised planning	Neglect	Markets	Asserting control	Rethinking
	Workforce governance responsibility	Department of Health	No identifiable organisation	Regional entities Employer-led	Ministry and various advisory entities	HWNZ, a dedicated HWP agency
	Impacts of reforms on policy and governance	Pre reform	Loss of structures and knowledge Use of advisory committees	Dispersal of governance to smaller operational entities	Fragmentation Duplication and ineffective responses	Consolidation Improved data management and integration Longer planning horizons
Implications for governance and planning	Planning practice	Medical manpower based	None observable	Employer-led planning, based on operational needs	Data gathering and situation analysis	Some planning re-centralisation Provide sector leadership Wider view of the planning function
	Planning concerns	Mal-distributions, Unsustainable delivery paradigms	Increasing visibility of workforce problems	No wider or longer view Shortages Rising dependence on overseas professionals and workers	Poor industrial relations Planning inefficiencies Data gaps	Incorporating the new vision Introducing team-based care Resistance to change Conservatism
Implications for methods	Principal methods	Stock and flow models Estimation of doctor numbers	No data available	Demand driven modelling Headcount based modelling	Aggregated demand models Improved data collection begins	Design thinking and workforce intelligence approach—integrated quantitative and qualitative data to meet future care scenarios and team-based workforces

Sources: [14, 20, 21]

In the 1970s and 1980s, health workforce planning was centralised and largely medically focussed. Overseen by a DoH committee, with New Zealand Medical Council input, it reported directly to the Minister of Health, with plans informed by stock and flow modelling [20]. The committee was conscious that medical manpower planning, i.e. doctor numbers, needed to be linked to the wider health workforce and acknowledged that productivity and mal-distribution improvement relied on introducing health team approaches as “in a publicly funded health service one of the few effective controls on the use of resources and the health expenditure is the number of doctors in the system” ([20], p.8).

However, the steady progress to establish consistent workforce governance faltered after the reform programme commenced in the late 1980s. This signals the next phase of health workforce policy, which began with “a decade of health sector reform and relative workforce neglect” ([21], p.105). Many health workforce structures and processes were lost in the DoH restructure as workforce planning was assumed by employing entities. By the mid-1990s, health workforce concerns had grown to the extent that the Minister of Health had established the Committee Advising on Professional Education (CAPE). Its report found inadequate attention was paid to health workforce issues due to a short-term financial and service efficiency focus. While the report proposed that a health education agency be established, this advice was not acted on. Thus, health sector employers continued to determine plans based on market needs [21], with the MoH encouraged to stay on this course [22]; employer-led planning continued into the 2000s.

By this time, persistent shortages of doctors and nurses had made the country increasingly reliant on overseas trained professionals [16]. Austere funding had affected the workplace escalating to industrial action [16], while tensions alienated clinical staff from DHB managers and both from government policy makers [11]. These problems re-emphasised the importance of health workforce development and its associated policies [23], and by the mid- to late 2000s, wage demands and training numbers had received some attention, though tensions remained [16].

In the mid-2000s, a number of health advisory committees [15] reported concerns over the ageing population and identified a range of cross-sectoral issues [21, 24, 25], indicating that little gain had been made since the CAPE report. Regulatory reform progress was forwarded though, with the passing of the 2003 Health Practitioners Competence Assurance Act, and while it continued to take a profession-based silo approach, it did provide the possibility for new roles and/or overlapping scopes of practice [26]. This led to the nurse practitioner role in 2004 [23, 27] and was followed up by further detailed workforce analysis [28].

While this progress was being made on understanding the health workforce environment, workplace conditions did not fare well. The health system continued to be troubled by mal-distribution and shortages, with specialist medical workforce projections predicting more of the same [29].

This situation reveals the risk of employer-led health workforce planning: that with the substantial activity comes fragmentation and duplication and an ever-increasing focus on operational efficiency. This was reflected by the DHBs’ plans tending to favour hospital and specialist care, with PC workforces not well catered for [15]. Despite this and without national supply monitoring, the MoH remained hopeful that solutions rested with workforce actor collaboration [30], though little success was forthcoming.

The late 2000s also saw a revised focus on medical education [30, 31], resulting in a new organisation providing oversight for New Zealand’s medical education and training [32, 33], shortly followed by another review recommending a substantial reconfiguration of the whole of health workforce’s planning and funding [26]. This review was opportune, as 2009 was a time of serious health workforce shortages [11]. The review also reiterated many previous messages about workforce integration and productivity [20], revealing the significant lack of workforce development progress over the past 30 years. The review noted that “a single agency, which has a whole of health and disability services workforce and whole of educational continuum responsibility, is needed if New Zealand is to have an affordable and fit-for-purpose health and disability services workforce” ([26], p.5).

Thus, late 2009 heralded the third phase of workforce policy, with Health Workforce New Zealand (HWNZ) being established as a National Health Board (NHB) business unit, situated within the MoH. HWNZ’s aim was to provide national leadership for the development of the country’s health and disability workforce and overall responsibility for planning and development of the health workforce to ensure that it is fit for purpose [34].

HWNZ implemented a workforce planning approach that embraced conditions of uncertainty [35] and began to develop health system intelligence capabilities to conceive new visions of health services and their workforces [35, 36] and realising these through a range of new initiatives. These initiatives included the Workforce Service Forecast (WSF), a clinician-led and patient-centred scenario, resulting in a forecast of future possible model(s) of care for a particular service aggregate [14], reducing the system’s reliance on profession-by-profession forecasting while accommodating inherent uncertainty and promoting emerging workforce and treatment innovations [34]. Other initiatives implemented by HWNZ were a comprehensive workforce forecasting model, incorporating variables such as workforce demographics, retirement patterns and immigration trends [37]; the use of more qualitative intelligence through scope of practice analysis [38] and a number of workforce innovation pilots [34, 39, 40].

In the late 2018, HWNZ faced administrative change. In response to the poor achievement of its principal objectives, the HWNZ Board was disestablished [41]. From a range of governance options, health workforce was made part of the MoH, headed by a Deputy Director General and supported by a non-executive advisory board [41]. Reflecting re-centralisation, this consolidation was seen as a means to mitigate HWNZ’s slow goal achievement progress, for while HWNZ was successful in health workforce intelligence gains, it was less successful at addressing workforce problems, creating a clear strategy and future pathways and providing sector leadership. These failings have been attributed to policy and accountability tensions due to HWNZ’s position as part of the NHB, while being reliant on MoH resources [42]. The fragmented nature of health care, the discipline-based lens’ of New Zealand’s workforce participants [21, 22, 30, 43] and their conflicting priorities [44] may also have contributed.

## Discussion and evaluation

The sequence of oscillating reforms created a number of issues for New Zealand’s health workforce. As outlined in Table 2, these issues can be arranged into three broad categories: (i) the implications stemming from the health reforms for the health workforce and service delivery, (ii) the implications for governance and their responses and (iii) the implications for planning methodologies.

### Implications for the health workforce and service delivery

New Zealand’s sequence of health system reforms were primarily focussed on gaining efficiency or value for money, changing institutional structures and funding mechanisms and introducing accountability through contracting. As a result, in the first 20 or so years of reform, there was little active health workforce policy. The decentralisation agenda eventuated in an employer-led focus which failed to deliver expected synergies, as it principally relied upon employers to take a view wider than their own interests. The resulting fragmentation and duplication, and workforce interventions were dominated by short-termism and crisis, leading New Zealand to become increasingly reliant on imported labour and skills to meet numbers gaps. A lesson from this is that a sole reliance on market forces to shape the workforce can lead to increasing inequalities in terms of workforce disposition and access for patients [35]. Moreover, as Imison et al. [45] observe, while employers may be better placed to assess and influence future demand, it is national bodies that are better placed to influence supply, a feature that was not readily recognised In New Zealand and was assumed to occur naturally. However, the resulting fallout was the initiator for HWNZ’s rethinking approach.

Though as observed in the United Kingdom, moving towards ever more centralised control may not necessarily result in successful integration and improved planning, rather it is suggested that integration should be based on common strands and their synergy [46]. The latter can be detected in HWNZ’s planning philosophy of patient-focussed future care scenarios built around teams, reflecting a shift in workforce frames of reference from a “do we have enough” frame towards “how can we more effectively deploy” [36].

Additionally, there is the issue of managing implementation, as the reforms did little to change the PC sector and its principal delivery model, the small independent practice. While PC sector capacity is being somewhat addressed by a policy preference for larger unified delivery units [12], the mal-distribution of GPs and uneven access to higher skilled nursing personnel is still particularly acute for rural areas and high-need populations. This presents problems for promoting a single care model across New Zealand and reflects the unlikelihood that single integration solutions exist [47], though a locally developed community-based chronic condition service model has been successfully implemented by smaller practice units [48]. Moreover, different reform policy prescriptions have been shown to have different consequences, providing indicators of what should be included when designing particular health reform agendas [6].

### Implications for governance and planning

The principal implication for governance is that more is needed than just a simple recasting of planning through more sophisticated supply-demand projections. The poor attention to the workforce over New Zealand’s reform period left recurring issues that tend to distract workforce planners from longer-term views. To overcome short-termism, there is the need for strengthening health workforce governance, which here means the policy development, education arrangements, regulatory oversight and collaboration structures, to enable New Zealand’s desired system goal of integration [19].

A proactive governance tool that has been offered as a means to achieve these types of outcomes is interprofessional education (IPE) [36]. IPE is an approach as part of medical and health worker training and professional development that provides improved collaborative practices, raises the confidence of individuals to work collaboratively [6], improves trust between providers and patients and facilitates workforce groups to engage collaboratively [49]. Educators, as they become more interprofessionally skilled, also contribute to improved student learning outcomes and experiences [50]. IPE has been found to positively influence health care human resource outcomes in terms of workplace quality, workforce factors such as recruitment, retention, turnover and satisfaction and acts to attract students and improve employment rates [51] and prepares health professionals for collaborative practice and clinical provision, which tends to benefit service provision [52].

Rees et al.’s [53] examination of health scenarios concluded that IPE was likely to improve the scenario outcomes and so has suggested assuring IPE access for a wide range of workforce roles as a future health workforce policy measure for New Zealand. In line with this, Fraher and Brandt [36] reflect that planners and educators should be collaborating to realise improved patient-focussed care supported by work-based learning. As such to improve integrated provision and workforces, it seems advisable that more attention be given to IPE in New Zealand’s health workforce planning and training mix.

Developing these governance capabilities and structures may require deeper and more analytical methods than simple stakeholder analyses to gather data for strategy and implementation [54]. Rees et al. [44] found that New Zealand’s workforce actors in different health sectors have divergent perceptions and priorities for workforce evolution, suggesting care should be taken when considering policy situations involving multiple actors.

Moreover, while the use of teams containing diverse staff and skills has been found to have a positive effect on care quality and patient satisfaction [55], there are traditions surrounding professional training, regulation and practise that result in institutional inertia preventing wider spread change [56]. It is here that attention needs to be paid to the roles of health workforce actors and targeting policy to lever effective system change [44]. Complementing this is continued to engagement with clinicians, educators and employers to achieve future care models [36] while recognising the limiting effects of professional resistance [38].

### Implications for planning methods and practices

The policy gap and lack of national workforce coordination resulting from the reforms provided HWNZ with an opportunity to re-envisage how workforces could be planned and to gather the supporting data to enable this. For instance, HWNZ’s planning approach is the only one so far reported to use design thinking [2]. The approach incorporates design thinking’s “creative human-centred problem solving approach that leverages empathy and collective idea generation” ([57], p.3) to develop solutions from the perspective of the user [58]. Along with the WSF process, HWNZ has been able to enact design thinking’s practice of reflection, analysis, visualisation and modelling [57, 58] into its planning [2].

While continuing to use traditional workforce forecasting methods, HWNZ has extended the range of tools that it has at its disposal. Their application has enabled the use of a wider range of planning methods to develop broader workforce intelligence variables [35, 36, 39, 40, 59, 60]. Over 2015/2016, the comprehensive workforce forecasting model was used to identify medical specialities’ ability to meet demand within the current model of health care, allowing for increased investments that aimed to reduce forecast shortages and mal-distributions [37]. HWNZ is also introducing more qualitative intelligence through scope of practice analysis. Scope overlap or plasticity analysis investigates the possible substitution of professionals at some stages of care [61, 62]. For example, Suter et al. [62] reviewed centralised intake processes and the roles, types and scopes of health care providers, physicians and support staff associated with the various steps to identify potential alternative providers. Likewise, HWNZ has reviewed plasticity for assessing general practice workloads as a means to have GPs concentrating on more complex cases in a scenario of comprehensive primary care delivered by integrated, multi-disciplinary teams [38]. HWNZ has also sought to drive workforce innovation and flexibility through a number of workforce innovation pilots. Evaluations of these indicate patient benefits and improved staff satisfaction, an introduction of specialist knowledge in to practice areas, improved teamwork potential and reductions of staff and system pressures [50, 52, 63–65].

However, HWNZ has also encountered some issues. For example, the introduction of the WSF process also had to deal with an existing predictive planning culture, which found participants to be “divided into those who believed the objective was to deliver a vision and a set of scenarios, and those who thought they were expected to develop a more detailed map of projected workforce numbers and composition” ([66], p. iv). Such discomfort with new planning methods or approaches is not unusual [67]. Even so, the WSF process was found to have been a successful means for bringing together interdisciplinary groups of professionals, to build capacity and to develop new ways of thinking about services and workforce plans [68]. The WSF’s common conclusions reinforce New Zealand’s health futures as integrated, valuing of self-care and reflective of community-based delivery [34].

Another issue relates to New Zealand’s slow pace of workforce change, with many of its innovation pilots failing to diffuse, as they remain embedded in the original site [26]. To improve workforce innovation diffusion, it has been recommended that the future projects should have evaluation as part of their designs and be better conceptualised with the “new roles [be] carefully defined in the context of the service and wider system” ([69], p.36). As such, HWNZ has identified that it needs to develop further capacity and capabilities for disseminating and embedding workforce innovation [34], though there are also positives. HWNZ’s application of design thinking in its workforce planning affords a possibility to support wider health policy implementation [57], through the emphasis on patient-centred care throughout the WSFs and the proposed use of plasticity models to assist with improved deployment of PC teams. Not only does this approach break with workforce planning’s limiting connection with the present’s institutional norms and infrastructures [12], it more readily aligns workforce planning with the New Zealand health strategy’s commitment to a more integrated care system [19].

These new methods also bring opportunities and contribute to strengthening governance. For example, the WSF process may serve as a model for DA service development as it is clinician led and involves a range of affected stakeholders. Specific sector plans have also been a direct result of the WSFs such as a renewed planning focus on community-based older person provision [35] and measures to improve home based care services [70, 71]. Plasticity modelling has similar potential for workforce allocation as it can clarify work force composition and reduce task transfer risks [61].

Though to more consistently achieve these outcomes, Rees et al. [72] point out that workforce planners should be embracing uncertainty and considering the more frequent use of complementary planning tools. This call may be met by some ambivalence or possibly resistance, as policy makers tend to be driven towards accuracy, rather than embracing uncertainty and ambiguity [67], and through unrealistic expectations of planning processes [45]. So, while this shift within New Zealand’s health planning and policy agencies would be desirable [72], it will likely take some time.

## Conclusion

This article has presented policy background to and the effects of persistent health reforms on New Zealand’s health workforce and its governance. Principally medically orientated pre to the late 1980s, the country’s workforce policy and planning was adversely affected by the extended period of reforms, leaving it with a short-run, operationally orientated employer-led focus. However, the advent of a centralised agency in 2009 offered an opportunity to rethink and to provide sector-wide policy coordination and a central point for workforce data gathering and analysis.

Importantly, the agency created for this task, HWNZ, reconsidered how health workforce planning may proceed and sought to understand how future services may be configured by applying a design thinking-influenced method to better respond to future health needs. The first step of this approach founded the use of WSFs, future scenarios of care developed by clinically led multi-skilled teams. Second, HWNZ has extended its concept of data to recognise qualitative workforce intelligence and is beginning to incorporate these to broaden its planning scope.

As such the case provides a lesson for other countries considering health reforms; it highlights the deleterious effects when the health workforce is not considered or included. But more than this, it also signals that providing the type of attention New Zealand has been able to give can revitalise workforce policy and governance, allowing the adoption of a wider range of perspectives, timeframes and planning tools to deal with increasing uncertainty and the move towards integrated services.

The policy challenge now confronting New Zealand’s health workforce is aligning governance with implementation. This requires locking in and building on changes at the policy and strategy levels, while continuing to develop the skills for engagement, collaboration and locally driven execution. It appears that a key question for New Zealand will be: how does it facilitate a transition towards the desired future health workforce, while maintaining appropriate numbers and skills to ensure health system functionality in the meantime?

## Data Availability

Not applicable

## Abbreviations

CAPE: Committee Advising on Professional Education
DA: District alliance
DHB: District health board
DoH: Department of Health
GP: General practitioner
HWNZ: Health Workforce New Zealand
IPE: Interprofessional education
MoH: Ministry of Health
NHB: National Health Board
PC: Primary care
WSF: Workforce Service Forecast
